# Demography and clinical pattern of newly diagnosed uveitis patients in Malaysia

**DOI:** 10.1186/s12348-022-00306-1

**Published:** 2022-09-01

**Authors:** Rajasudha Sawri Rajan, Shelina Oli Mohamed, Mohamad Aziz Salowi, Malaysian Uveitis Study Group, Shakira Jeffrey, Farah Ibtisam Ibrahim, Wan Dalila Wan Hazmy, Vinuthinee Naidu Munisamy Naidu, Josephine Lee Oon Hui, Nurul Ashikin Abdullah, Nur Hafidza Asiff, Mohd Faizal Haron, Caroline Binson, Tan Li Mun, Niki Ho, Nazima bt Shadaht Ali, Muhammad Najmi Khairuddin, Chong Ka Lung, Izzati Othman, Matthew Tong Jong Haw, Abdul Hadi bin Rosli, Siti Ilyana Binti Ghani, Zunaina Embong, Chiang Wai Seng, Viona Lai Mi Shan, Teh Wee Min, Lee Wei Wei, Justin Yeak Dieu Siang, Tajunisah Iqbal, Nurul Fatin Fadzlullah Shuhaimi, Indira Nadras, Chan Li Yen, Melinder Kaur, Noor Widyani, Chiang Wai Seng, Rosiah Muda, Mandy Cheong Moon Yee, Hafiza Yaacob, Tengku NorinaTengku Jaffar, Abdah Azimah, Foo Lee Min, Norazizah Mohd Amin, Krishnalatha Buandasan, Aasiah Ahmad Sharifuddin, Atikah Asini, Gayathri Govindasamy, Hayati Abd Aziz, Krishnadevi Thiyagarajam, Goh Siew Yuen, Muhammad Yusuf Abdurrahman, Nandini Vijaya Singham, Sangeeta Kuganasan, Wan Mohd Hafidz Wan Abd Rahman, Patricia Ann, Noor Khairul Rasid, Ng Hui Ruan, Goh Chon Han, Ngim You Siang, Siti Farhah Adilah, Hamisah Ishak, Nor Azita Ahmad Tarmidzi, Kenneth Teow, Mazaya Mahmud, Lim Say Suan, Seow Sieng Teng, Nor’ain Md Rawi, Zalifah Zakiah, Zairah Zainal Abidin, Mohamad Israk, Rozita Ismail, Rafidah Saleh, Zalilawati Mohmad, Thye Ghee Yeap, Safiyah Jameelah Mohd Yusof, Azie Anne, Munira Yusoff, Norwazilah Mohd Ansul, Kenneth Lee, Chieng Lee Ling, Tang Pui Ling, Yushaniza Yaacob, Hurul Aini, Tara George, Norazizah Mohd Amin, Ministry of Health Malaysia

**Affiliations:** 1 Medical Retina and Uveitis Department, Hospital Selayang, Batu Caves, Malaysia; 2 Medical Retina and Uveitis Department, Hospital Shah Alam, Shah Alam, Malaysia; 3 Public Health Department, Hospital Selayang, Batu Caves, Malaysia

**Keywords:** Anterior uveitis, Epidemiology, Infectious uveitis, Incidence, Intermediate uveitis, Malaysia, Panuveitis, Posterior uveitis, Uveitis

## Abstract

**Introduction:**

Uveitis is one of the common causes of visual impairment in Malaysia. It remains a challenging entity to diagnose and manage due to variation in its clinical presentation. This study aims to observe the demographic and clinical pattern of cases from the participating ophthalmology units in Malaysia.

**Methods:**

This study involved prospective and multicentered data collection for patients newly diagnosed with uveitis from 1^st^ January 2018 to 31^st^ December 2018. Variables collected and analyzed included age, gender, ethnicity, nationality, state of origin, laterality, granulomatous or non-granulomatous uveitis, and etiology of uveitis.

**Results:**

A total of 1199 newly diagnosed uveitis patients were analyzed within the study period. There was a significant association between the anatomical location of uveitis with age at presentation. The percentage of patients with anterior uveitis was higher in the ‘40 to 60’ years and ‘above 60’ years age groups at 52.1% (*n* = 210) and 61.3% (*n* = 114) respectively. In contrast the percentage of patients with posterior and panuveitis was higher in the 1 to 20 and 20 to 40 years age groups at 51.4% (*n* = 54) and 48.7% (*n* = 246) respectively.

Sixty three percent of the patients presented with unilateral uveitis (*n* = 760, *p* < 0.001) vs bilateral. Non-granulomatous uveitis comprised 84.5% of all patients (*n* = 1013, *p* < 0.001) compared to granulomatous uveitis. Non-infectious etiology contributed to 65.7% of all patients (*n* = 788, *p* < 0.001) with the majority being unclassifiable uveitis (*n* = 686, 57.2%,). Specific inflammatory entities contributed to only 8.5% (*n* = 102) of the non-infectious causes with Vogt-Koyanagi-Harada (VKH) syndrome being the most common (*n* = 25, 2.1%,). Infectious uveitis comprised 34.3% (*n* = 411) with tubercular (TB) uveitis (*n* = 105, 8.8%) and viral uveitis (*n* = 107, 8.9%) contributing the most followed by ocular Toxoplasmosis (*n* = 93,7.8%).]

**Conclusion:**

This study has highlighted the demographic data and common causes of uveitis in Malaysia.

## Introduction

Uveitis is one of the causes of visual impairment globally. It is estimated to contribute to at least 10% of blindness worldwide [1]. Although it is not the main contributor to blindness such as cataract, Age-related macular degeneration (ARMD), and glaucoma, it can cause blindness primarily through the disease process itself or via secondary complications such as cataract and glaucoma. This makes optimal management of uveitis an important aspect of eye care. However, one of the main challenges of uveitis management is reaching an accurate diagnostic conclusion at the onset. Over time, the evolvement and advancement of diagnostic tools and imaging techniques have enhanced the way we diagnose and manage uveitis patients [2]. As the common causes of uveitis vary worldwide, the incidence for certain uveitides may differ according to region [3–9]. Understanding the epidemiology and causes of uveitis in various regions will further aid clinicians in a targeted approach to managing patients with diagnostically challenging uveitis.

This study was carried out to ascertain common causes of uveitis in Malaysia. As Malaysia is a diverse and multi-ethnic nation, there is further intrigue in determining the demography of our patients diagnosed with uveitis.

## Methodology

This was a prospective, multicentered study carried out in numerous ophthalmology units around Malaysia. New patients in these centres diagnosed with uveitis from 1^st^ January 2018 to 31^st^ December 2018 were enrolled into the study. A total of 1522 patients who had completed diagnosis and work up were recruited. These patients from all 14 states in Malaysia were recruited and diagnosed by Ophthalmologists. These centres had an Ophthalmology Department with the necessary equipment to diagnose and manage uveitis patients. All of these patients were seen and enrolled during the first visit.

Out of these 1522 patients, only 1199 patients from 40 centres were analyzed. The exclusion criteria were incomplete data, absence of final diagnosis, endophthalmitis including endogenous in origin, scleritis, peripheral ulcerative keratitis and uveitis secondary to surgery, lens or trauma.

Excel sheets with all the required variables were distributed to the participating centres to ensure standardized data collection across all hospitals. Each centre would have an Ophthalmologist appointed to review potential patients, collect and enter the data into the excel sheet provided. Any inquiries regarding data collection and data entry would be addressed by the principal investigators remotely and in a confidential manner.

Demographic data obtained included age, gender, ethnicity, nationality, state of origin, laterality, granulomatous or non-granulomatous uveitis, and etiology of uveitis.

The anatomic classification of uveitis was followed based on SUN (Standardization of Uveitis Nomenclature) Working Group criteria [10]. The four main classifications were anterior uveitis, intermediate uveitis, posterior uveitis and panuveitis. Patients diagnosed with neuroretinitis were included in the posterior uveitis classification.

All recruited patients were consented for complete ophthalmic assessment including vision, intraocular pressure measurement (IOP), slit lamp assessment and the necessary diagnostics at their respective centers. The diagnostic modalities used depended on the availability of these instruments at the centers. These included Fundus Photography, Anterior Segment photography, OCT (Optical Coherence Tomography), Fundus Fluorescein Angiography with or without Indocyanine Green Angiography and B-scan Ultrasound where necessary.

All patients that required further laboratory investigation would receive a basic uveitis blood panel and radiographic work up. This would include Full Blood Count (FBC with differentials), ESR (erythrocyte sedimentation rate) + /CRP (C-reactive protein), Mantoux test, VDRL (Venereal Disease Research Laboratory)/RPR (Rapid Plasma Reagin) test and TPHA (Treponema Pallidum Hemagglutination Assay) test for syphilis, and chest radiography. Other tests were more targeted, based on relevant history and clinical findings. These included *Toxoplasma gondii* antibodies (IgM and IgG), TB QuantiFERON Gold (if a second confirmatory tuberculous test was required), HLA-B27 haplotyping, specific infectious testing such as for Leptospirosis, Melioidosis, and Bartonellosis, viral aqueous tap PCR for *Cytomegalovirus* (CMV)/*Varicella -zoster virus* (VZV) and *Herpes Simplex Virus* (HSV), ANA (Antinuclear Antibody), ANCA (Anti-Neutrophil Cytoplasmic Antibodies) and other vasculitides work-up when required.

Patients were diagnosed based on clinical findings and relevant positive laboratory tests. Patients diagnosed with Behcet’s Disease had to fulfil the criteria as determined by the International Study Group for Behcet’s Disease [11]. Cat scratch disease diagnosis was made based on ocular manifestation and a least one positive laboratory test. They must be positive for anti *Bartonella henselae* IgM ± IgG antibodies detected and/or PCR positive for *B.henselea*DNA if enlarged lymph nodes were present [12]. Toxoplasma uveitis was diagnosed based mostly on clinical findings and at least one positive serology IgG ± IgM for *Toxoplasma gondii *[13].

For the diagnosis of tubercular uveitis, we used the recommendations as by the Collaborative Ocular Tuberculosis Study group (COTS) for diagnosis[14]. This would include clinical features suggestive of ocular tuberculosis with at least a positive Mantoux test (> 10 mm) or positive QuantiFERON gold test if a confirmatory test such as isolation of *M.tuberculosis* from tissue or bodily secretion was not available. Leptospira uveitis was diagnosed based on history of contact and exposure as well as *microscopic agglutination test*(MAT) which is the gold standard in Malaysia [15, 16]. The diagnosis of Primary Vitreo-retinal Lymphoma (PVRL) was made from the positive cytology results of the vitreous biopsy.

Unclassifiable uveitis was classified as such if there was no identifiable ocular entity and no associated systemic condition to explain the cause of the uveitis, and the full uveitis work up in these group of patients revealed normal or negative results. This would also include entities that could not be classified into specific idiopathic causes such as AZOOR, Behcet’s Disease and such [17, 18].

The data obtained were analyzed using SPSS software version 25.0 (SPSS Inc.,Chicago, IL).The variables were analyzed using the Chi-Square test. Multiple logistics regression analysis was used to determine association between infectious causes and its variables. *P-*value of < 0.05 was considered to be statistically significant.

The study adhered to the tenets of the Declaration of Helsinki. The study was registered with the national NMRR (National Medical Research Registry).

## Results

Out of 1522 patients enrolled in this study, 1199 newly diagnosed uveitis patients from the 40 centers were analyzed after fulfilling the inclusion and exclusion criteria. The demographic data is shown below (Table 1).

**Table 1:** Demographic Data and Clinical Features according to SUN classification

	Total cases (*N* = 1199)	Anterior Uveitis	Intermediate Uveitis	Posterior Uveitis	Panuveitis	*P* value
		(*N* = 560)	(*N* = 136)	(*N* = 249)	(*N* = 254)	
	*n (%)*	*n (%)*	*n (%)*	*n (%)*	*n(%)*	
Age						
Mean (SD)	41.4(16.36)	44.9(16.47)	39.7 (16.08)	36.5(15.55)	39.6(15.45)	< 0.001^a^
Age group						
1 to 20	105 (8.8)	39 (37.1)	12 (11.4)	31 (29.5)	23 (21.9)	< 0.001^b^
20 to 40	505 (42.1)	197 (39.0)	62 (12.3)	130 (25.7)	116 (23.0)	
40–60	403 (33.6)	210 (52.1)	45 (11.2)	63 (15.6)	85 (21.1)	
> 60	186 (15.5)	114 (61.3)	17 (9.1)	25 (13.4)	30 (16.1)	
Gender						0.314^b^
Male	622 (51.9)	279 (44.9)	68 (10.9)	131 (21.1)	144 (23.2)	
Female	577 (48.1)	281 (48.7)	68 (11.8)	118 (20.5)	110 (19.1)	
Race						0.002^b^
Malay	736 (61.4)	333 (45.2)	77 (10.5)	173 (23.5)	153 (20.8)	
Chinese	183 (15.3)	97 (53.0)	18 (9.8)	30 (16.4)	38 (20.8)	
Indian	140 (11.7)	79 (56.4)	22 (15.7)	16 (11.4)	23 (16.4)	
Others	138 (11.5)	51 (37.0)	18 (13.0)	30.0(21.7)	39 (28.3)	
State of Origin						< 0.001^b^
Central (Selangor, KL, Putrajaya)	473 (39.4)	215 (45.5)	66 (14.0)	90 (19.0)	102 (21.6)	
Nothern (Perak, Kedah, Perlis, Penang)	261 (21.8)	135 (51.7)	12 (4.6)	61 (23.4)	53 (20.3)	
East coast (Terengganu, Kelantan, Pahang)	151 (12.6)	67 (44.4)	20 (13.2)	37 (24.5)	27 (17.9)	
Southern (Johor, Melaka, Negeri Sembilan)	159 (13.3)	87 (54.7)	19 (11.9)	28 (17.6)	25 (15.7)	
East Malaysia (Sabah, Sarawak)	153 (12.8)	54 (35.3)	19 (12.4)	33 (21.6)	47 (30.7)	
Nationality						0.667^b^
Local	1147 (95.7)	540 (47.1)	129 (11.2)	236(20.6)	242 (21.1)	
Foreigner	52 (4.3)	20 (38.5)	7 (13.5)	13 (25.0)	12 (23.1)	
Hypopyon						
Present	65 (5.4)	42 (64.6)	8 (12.3)	2 (3.1)	13 (20.0)	0.002^b^
Absent	1132 (94.4)	518 (45.8)	127 (11.2)	246(21.7)	241 (21.3)	
Laterality						< 0.001^b^
Unilateral	760 (63.4)	424 (55.8)	71 (9.3)	151(19.9)	114 (15.0)	
Bilateral	328 (27.4)	87 (26.5)	60 (18.3)	73 (22.3)	108 (32.9)	
Granulomatous						< 0.001^b^
Granulomatous	166 (13.8)	64 (38.6)	18 (10.8)	16 (9.6)	68 (41.0)	
Non-granulomatous	1013 (84.5)	496 (49.0)	116 (11.5)	216(21.3)	185 (18.3)	
Etiology						< 0.001^b^
Infectious	411 (34.3)	82 (20.0)	38 (9.2)	170(41.4)	121 (29.4)	
Non-infectious (Unclassified & Inflammatory)	788 (65.7)	478 (60.7)	98 (12.4)	79 (10.0)	133 (16.9)	

^a^One-way ANOVA Test

^b^Chi-Square Tests

There were 622 males (51.9%) and 577 females (48.1%) with male to female ratio of 1.07:1. The age range was from 9 to 88 years old. The mean age was 41.4 (SD: ± 16.36 years). The highest number of patients in an age group was from 21–40 years old accounting for 505 patients (42.1%), and the lowest was 20 years and younger age group accounting for 105 patients (8.8%). Majority of the patients (*n*=736, 61.2%) were Malay in origin.

The hospitals were analyzed according to their geographical zones. The highest number of patients were from Central Malaysia with 473 patients (39.4%). The central zone of Malaysia is the most urbanized area in Malaysia consisting of Kuala Lumpur, Selangor and Putrajaya. The least number of patients were from the East Coast Malaysia (Kelantan, Pahang and Terengganu) with 151 patients (12.6%). A total of 1147 patients were residential Malaysians (95.7%). The remaining 52 patients (4.3%) were residents of other nationalities.

With regards to the anatomical classification, anterior uveitis (AU) was seen in 46.7% (*n* = 560), followed by panuveitis (PANU) at 21.2% (*n* = 254), posterior uveitis (PU) 20.8% (*n* = 249) and intermediate uveitis (IU) at 11.3% (*n* = 136).

There was a significant association between the anatomical location of uveitis with age at presentation. Anterior uveitis was seen in 560 patients (46.7%). The percentage of patients with anterior uveitis was higher in the 40 to 60 years and above 60 years age groups at 52.1% (*n* = 210) and 61.3% (*n* = 114) respectively. In contrast the percentage of patients with posterior and panuveitis was higher in the 1 to 20 and 20 to 40 years age groups at 51.4% (*n* = 54) and 48.7% (*n* = 246) respectively.

A unilateral presentation was noted in 760 patients (63.4%) vs bilateral presentation in 328 patients (27.4%). Most cases presented with non-granulomatous uveitis (*n* = 1013, 84.5%) as opposed to granulomatous uveitis (*n* = 166, 13.8%). Hypopyon was present in only 65 patients overall (5.4%).

Overall, non-infectious uveitis (inflammatory and unclassified) was seen in 65.7% (*n* = 788) and infectious uveitis comprised of the remaining 34.3% (*n* = 411).

The causative pattern of uveitis differed between all four anatomical locations. (Table 2).Out of 560 cases of AU, 434 (77.5%) were unclassifiable. Viral uveitis comprised of 58 (10.9%) cases, presumed tubercular uveitis was seen in 20 (3.6%) and there were 3 cases (0.5%) of syphilitic anterior uveitis. As for non-infectious etiologies, HLA-B27 related anterior uveitis was diagnosed in 2.7% (*n* = 15) of the cases while there were 4 cases (0.7%) each of Juvenile Idiopathic Arthritis (JIA) -related anterior uveitis and Fuchs Heterochromic Iridocyclitis (FHI) respectively.

**Table 2:** Causative Pattern of New Diagnosed Uveitis according to SUN classification

Causes	Total	Anterior Uveitis	Intermediate Uveitis	Posterior Uveitis	Panuveitis
Unclassifed	686	434	88	73	91
TB	105	20	17	31	37
Syphilis	53	3	15	7	28
Viral uveitis	107	58	0	34	15
Toxoplasmosis	93	0	0	58	35
Bartonella	29	0	0	29	0
Sarcoidosis	11	0	6	0	5
Behcets	15	0	3	1	11
VKH	25	0	0	1	24
HLA B27	15	15	0	0	0
Other infectious	18	0	4	8	6
Other Inflammatory	31	23	2	6	0
Primary VR Lymphoma	4	1	1	0	2
Systemic Vasculitides	7	6	0	1	0
Total	1199	560	136	249	254

Intermediate uveitis (IU) was diagnosed in 136 cases with 64.7% (*n* = 88) being unclassifiable. Presumed tubercular and syphilitic etiologies formed 12.5% (*n* = 17) and 11.0% (*n* = 15) of the cases respectively. Non-infectious etiologies were Sarcoidosis (*n* = 6, 4.4%), Behcet’s Disease (*n* = 3, 2.2%) and Multiple Sclerosis (MS) (*n* = 1, 0.7%).

Posterior uveitis formed 249 of the cases out of which 73 (29.3%) were unclassifiable. Infectious entities were the predominant etiology in 167 (67.1%) of the cases. The main infectious causes were Toxoplasmosis (*n* = 58, 23.3%), viral uveitis (*n* = 34, 31.8%), presumed tubercular uveitis (*n* = 31, 12.5%), Bartonellosis (*n* = 29, 11.6%) syphilitic (*n* = 7, 2.8%) and other infectious entities (*n* = 8, 3.2%) (Meliodosis (*n* = 4), Leptospirosis (*n* = 1), DUSN (*n* = 1), Toxocara (*n* = 2)).

Panuveitis comprised of 254 cases. Non-infectious entities included VKH ( *n* = 24, 9.4%), Behcet’s disease (*n* = 11, 4.3%) and Sarcoidosis ( *n* = 5, 2.0%). Infectious entities were presumed tubercular uveitis (*n*=37, 14.6%), syphilis (*n*=28, 11.0%), Toxoplasmosis (n=35, 13.8%) and viral uveitis (n=15, 5.9%). 

Overall, unclassifiable uveitis was the most common aetiology diagnosed in 686 (57.2%) out of the 1199 patients. Out of the remaining 42.8% (*n* = 513), 405 ( 33.8%) were infectious in origin. These included viral uveitis ( CMV, VZV, HSV) at 8.9% (*n* = 107) followed by tubercular uveitis at 8.8% (*n* = 105), Toxoplasmosis at 7.8% (*n* = 93), syphilitic uveitis with 4.4% (*n* = 53) and Bartonellosis at 2.4% (*n* = 29). Non-infectious etiologies contributed to the remaining 108 cases which included VKH (*n* = 25, 2.1%), Behcets disease (*n* = 15,1.3%), HLA-B27 (*n* = 15,1.3%) and Sarcoidosis (*n* = 11,0.9%).

Patients with infectious uveitis were also analysed in comparison to patients with non-infectious uveitis (Table 3). Differences in age groups, ethnicities, state of origin and classification of uveitis were statistically significant between the two groups. In all age groups, ethnicities and state of origins, the non-infectious causes were predominant.

**Table 3:** Features of Newly Diagnosed Uveitis with Infectious vs Non- Infectious Etiologies

Variables	Infection		*p*-value^a^	OR (95% CI)	*p*-value^b^
	Infectious	Non-infectious			
	n (%)	n (%)			
Age Group					
< = 20	46 (50.5)	45 (49.5)	< 0.001	1.29 (0.74, 2.23)	0.367
21—40	208 (48.1)	224 (51.9)		1.34 (0.96, 1.88)	0.090
41 – 60	123 (34.5)	234 (65.5)		1.00 (ref)	
> 60	59 (36.9)	101 (63.1)		1.51 (0.97, 2.36)	0.071
Race					
Malay	288 (45.6)	343 (54.4)	0.001	1.76 (1.08, 2.87)	0.023
Chinese	60 (35.9)	107 (64.1)		1.26 (0.71,2.25)	0.428
Indian	34 (27.9)	88 (72.1)		1.00 (ref)	
Others	53 (44.5)	66 (55.5)		1.45 (0.73, 2.87)	0.286
Gender					
Male	247 (45.2)	299 (54.8)	0.023	1.34 (1.00,1.78)	0.051
Female	189 (38.3)	305 (61.7)		1.00 (ref)	
State					
Central	188 (45.9)	222 (54.1)	0.001	2.54 (1.58, 4.10)	0.000
Northern	97 (43.9)	124 (56.1)		2.21 (1.30, 3.74)	0.003
East Coast	54 (42.5)	73 (57.5)		1.65 (0.91, 2.97)	0.097
Southern	39 (26.4)	109 (73.6)		1.00 (ref)	
East Malaysia	58 (43.3)	76 (56.7)		1.61 (0.85, 3.03)	0.142
Anatomical Classification Uveitis					
Anterior Uveitis	97 (19.5)	401 (80.5)	< 0.001	1.00 (ref)	
Intermediate Uveitis	44 (37.9)	72 (62.1)		2.53 (1.61, 3.99)	0.000
Posterior Uveitis	157 (77.7)	45 (22.3)		14.31 (9.45,21.66)	0.000
Panuveitis	138 (61.6)	86 (38.4)		6.52 (4.54, 9.36)	0.000
Hypopon					
Absent	418 (42.5)	565 (57.5)	0.089		
Present	17 (30.9)	38 (69.1)			
Granulomatous Status					
Granulomatous	62 (45.6)	74 (54.4)	0.278		
Non-granulomatous	362 (40.7)	528 (59.3)			

^a^Chi-square test

^b^Multiple Logistic Regression

There was a significant association between infectious uveitis with anatomical classification of uveitis (*p* < 0.001). Posterior uveitis (OR 12.54, 95% CI 8.81–18.13) and panuveitis (OR 5.34, 95% CI 3.78,7.55) were more likely to be infectious.

## Discussion

There are multiple region-oriented studies carried in other parts of the world to determine prevailing pattern of uveitis [3–9]. For Malaysia, this would be the first such study that has been conducted nationwide to offer us some insight into the causes and diagnostic pattern of uveitis in Malaysia.

This study reflects the real-life scenario in the management of uveitic patients nationwide especially in general hospitals. Almost all the secondary and tertiary hospitals in the country have a dedicated ophthalmology department equipped with the necessary facilities to carry out uveitis consultations independently. In the event when an expert consultation is required, the general ophthalmologists would liase with the uveitis team in tertiary centres like Hospital Shah Alam, Hospital Selayang or Hospital Kuala Lumpur. They would either carry out a teleconsultation if the patients were remote or incapacitated, or transportation would be arranged to one of these bigger hospitals for a face-to-face consultation. Alternately, visiting consultations are also carried out in certain centers where the number of uveitis patients are high. Therefore, the diagnosis of these patients is usually comprehensive with the support from the local uveitis specialists.

The gender and ethnic population in our study is reflective of the country’s population. According to the United Nation’s Department of Economic and Social Affairs, in 2020 the population of Malaysia stands at 32.37 million. The Malaysian-Malays are the highest with 69%, followed by Malaysian-Chinese with 22.5%, Malaysian-Indians with 6.8%, and the smaller indigenous groups constituting around 1.7%. Men constitute around 50.7% of the population and this is reflective in our study as well.

It is not surprising that most of our patients are from the more densely populated, urbanized states, as infrastructure and ease of transportation play a crucial role in seeking healthcare in Malaysia. It is also of note that 4.4% of our patients were not Malaysians. It is likely that with urbanization and migration into the developing Malaysia, more non-Malaysians are seeking care in our hospitals.

We also note that roughly one third of our patients have an infectious etiology. These numbers correlate with many other uveitis epidemiological studies [19]. When compared with the meta-analysis study carried by Tsirouki et el, certain infectious etiologies correspond with causes in fellow Asian countries. In our study, viral entities contributed to 8.9% of all etiologies. This would include herpetides such as aqueous viral PCR (CMV,VZV,HSV) positive anterior uveitis, Acute Retinal Necrosis (ARN), and Cytomegalovirus (CMV) Retinitis. This is similar with a Singaporean study that showed incidence of herpetic uveitis to be 9.2% [8, 20].

Tubercular uveitis is equally high in our study (8.8%). This would correlate with TB being an endemic communicable disease in Malaysia. In 2018, a total of 25,173 patients were diagnosed with TB with an estimated incidence rate of 92 cases per 100,000 population [21]. In Tsirouki’s paper, the countries high with TB uveitis are India (10.1%), Iraq (11.4%) and Saudi Arabia (10.8%).

The other common infectious causes would seem to be related to transmission by cats such as toxoplasma uveitis which accounts for 7.8% of cases and bartonella uveitis (neuroretinitis) which accounts for 2.5%. This would contribute to almost 10% of infectious causes among the patients in the study. As there are lack of data on ocular toxoplasmosis, ocular bartonellosis or systemic bartonellosis in Malaysia, in this study we assume cat-related infectious diseases are reasonably common as cats are the predominant domestic pet in Malaysia [22].

We also noticed in our study that posterior and panuveitis were more likely to be infectious as compared to anterior and intermediate uveitis. However, in our clinical practice, patients who present with anterior uveitis are not as urgently investigated for infectious causes if they are not severe. Therefore, it is possible there is under-reporting of infectious causes in this group. Nevertheless, this reinforces the fact that infectious causes remain an important aetiology of uveitis among Malaysians sometimes rendering management of the patients more complex. Posterior uveitis in particular has a higher probability of being infectious. 

It should also be noted that in our study, we excluded patients diagnosed with endophthalmitis, including endogenous endophthalmitis. Although some of these patients had infective causes like Klebsiella, as they were systemic in nature and the ocular inflammation was presumed based on clinical findings and indirect culture yield such as blood culture and not direct yield such as ocular fluids, we decided to exclude them from our study.

More than half of our patients’ etiology was presumed to be idiopathic or in our study we named them unclassifiable uveitis. Worldwide, this appears to be fairly similar [6–9, 24, 25]. However, the unclassifiable uveitis cases in this study may include truly idiopathic uveitis as well as patients that have had thorough investigations but only within the capacities of the respective centres. Furthermore, considering the diagnosis was made mostly by general ophthalmologists, certain specific inflammatory or infectious entities may have been under diagnosed [21].

Among the non-infectious causes, Vogt-Koyanagi-Harada appears to be the most prevalent cause, followed by Sarcoidosis, Behcet’s Disease and spondyloarthropathy-associated uveitis. The reason these uveitides may not be as numerous compared to other studies could again be related to lack of experience and knowledge by the primary treating Ophthalmologist. Furthermore, obtaining HRCT Thorax, ACE serology, HLA B-5, HLA B-55, HLA B-27 may not be easy as these tests are not readily available.

It is also worthwhile to note that a significant number of Malaysians seek treatment in the private healthcare sector, thereby potentially contributing to an under reporting of uveitis cases in this study.

In Malaysia, diagnosing and treating patients with uveitis remains a challenge in some ways. The diagnostic challenges are primarily due to lack of resources and uveitis expertise in many far-out hospitals. Equipment such as OCT, Angiography and B-scan ultrasonography (USG) may be lacking in departments that are smaller and remote compared to the centers in the bigger towns and cities. There are also certain tests that are hard to obtain such as the viral PCR tests in these places. There are other specific tests that are easily available but the cost remains a barrier for most patients seeking treatment such as the highly pertinent but costly test, TB QuantiFERON Gold/(Interferon Gamma Release Assay)IGRA tests for TB. Due to these many issues, sometimes arriving at the final diagnosis may be difficult.

## Conclusion

In conclusion, this study from Malaysia revealed that non-infectious uveitis was predominant out of which more than half were categorised as unclassifiable uveitis. Vogt Koyanagi- Harada, Behcet’s Disease and HLA B-27 were the most common inflammatory entities. Infectious uveitis contributed to a significant number of patients, with viral tveitis, tubercular uveitis and Toxoplasmosis being the most common aetiologies. Anterior uveitis was most common followed by panuveitis and posterior uveitis. Posterior uveitis and panuveitis were more likely to be infectious although this may be due to under reporting of infectious anterior uveitis. The significantly lower number of specific inflammatory entities reported in this study may be related to  under-diagnosis and challenges faced from various other factors such as geographic distribution, logistics and access to diagnostic tools, testing centres or reagents.

## Data Availability

The datasets used and/or analysed during the current study are available from the corresponding author on reasonable request.
